# Immunostaining of P450c17, Aromatase and Oestrogen Receptor Alpha in Germ and Somatic Cells During Gonadal Development in Greater Rhea

**DOI:** 10.1111/ahe.70091

**Published:** 2026-02-13

**Authors:** Marilú Cristofoli, Danielle Cristina Calado de Brito, Mayla Magalhães de Oliveira Alcobaça, Flavia Maria Pia Montenegro Donoso, Fabiana Morse Gosson Jorge, Marco Aurélio Schiavo Novaes, Moacir Franco Oliveira, Antônio Chaves de Assis Neto

**Affiliations:** ^1^ School of Veterinary Medicine and Animal Science University of São Paulo São Paulo Brazil; ^2^ Escuela de Medicina Veterinaria, Unidad de Anatomía Veterinaria, Facultad de Recursos Naturales y Medicina Veterinaria Universidad Santo Tomás Santiago Chile; ^3^ University of International Integration of Afro‐Brazilian Lusophony – UNILAB Redenção Brazil; ^4^ Department of Animal Science Federal Rural University of Semiarid ‐ UFERSA Mossoró RN Brazil

**Keywords:** avian embryogenesis, gonadogenesis, hormonal regulation, ratite, sexual differentiation, steroidogenesis

## Abstract

The greater rhea ( *Rhea americana* ), the largest bird species in South America, has significant economic value due to its meat, eggs, leather, feathers and oil. However, its lack of external sexual dimorphism poses challenges for reproductive management and breeding programmes. Key factors involved in avian gonadal differentiation include the cytochrome P450c17 (CYP17A1), P450 aromatase and the oestrogen receptor alpha (ERα). This study investigated the spatiotemporal immunolocalization of these proteins in germ and somatic cells of gonadal crests and developing gonads in *R. americana* embryos from Day 9 to Day 24 of incubation. Immunohistochemistry revealed consistent expression of all three markers throughout gonadogenesis, with distinct intensity patterns between sexes and cell types. P450c17 has been detected in both germ and somatic cells in testes and ovaries, with increasing expression from the onset of sexual differentiation. Aromatase and ERα were strongly expressed in the ovarian cortex and lacunar regions, and moderately present in testicular cords and interstitium. These findings suggest coordinated endocrine signalling between germ and somatic cells during embryonic sexual differentiation in *R. americana* and provide foundational insight for future research on reproductive development and sex control in ratites.

## 1 Introduction

The rhea ( *Rhea americana* ) is a bird native to South America, belonging to the order Rheiformes and the family Rheidae, and is endemic to Brazil, Paraguay, Uruguay and Argentina (Picasso and Mosto 2016; Sick 2001). This bird is unable to fly and is therefore included in the ratite group. In addition, it is a species widely bred in captivity due to its zootechnical potential, providing meat, eggs, leather, feathers and oil, showing adaptability and economic relevance (Almeida 2003). Although they are easily bred alongside other commercial breeds, studies on their reproductive biology are still quite scarce.

Given the lack of apparent sexual dimorphism, the study of embryonic development, with an emphasis on gonadal differentiation and hormonal regulation, is essential for understanding the mechanisms that govern sexuality in this species (Almeida 2003; Almeida et al. 2015). Our group recently developed the first detailed description of gonadal development in rheas (on Days 9, 12, 15, 18, 21, 24 of incubation), identifying the morphological window of sexual differentiation around Day 15 of incubation (Cristofoli et al. 2025). In chickens, a more detailed description of the complete period of embryonic development (21 days) was made by Hamburger and Hamilton (1992), which is why most of the studies dealing with sexual differentiation and gonadal development in birds are in this species.

In addition, with the marked development of the poultry sector, the steroidogenic and hormonal cascades during embryonic development should also be considered relevant in order to improve management conditions. It is well known that the reproductive endocrine system is regulated by a complex network of hormonal interactions that control gametogenesis, gonadal function and fertility (Bruggeman et al. 2002; Estermann et al. 2020). Two important proteins in this system include the aromatase enzyme (P450), the cytochrome P450 enzyme 17α‐hydroxylase (P450c17) and the steroid hormone 17β‐estradiol (E2); these factors not only regulate ovarian and testicular function in a coordinated manner, but are also fundamental for sexual differentiation in birds (Smith and Sinclair 2004; Estermann et al. 2021).

The enzyme P450c17 is involved in steroid biosynthesis, influencing androgen production by cleaving progesterone into androstenedione (Bruggeman et al. 2002; Nakabayashi et al. 1998; Burris‐Hiday and Scott 2021). In *Gallus* sp., the expression of P450c17 enzyme mRNA and the production of androgens are essential for the development of the male gonad during embryonic development, as is the absence of P450 aromatase and estradiol (E_2_) synthesis (Nomura et al. 1999; Payne and Hales 2004; Yoshida et al. 1996; Rosati et al. 2021). The aromatase enzyme is responsible for converting androgens (androstenedione and testosterone) into oestrogens (estrone and E_2_) (Rosati et al. 2020; Simpson and Davis 2001). Studies show that P450 and CYP17 enzyme transcripts are already present in the gonads of avian embryos even before gonadal differentiation (Bruggeman et al. 2002; Nomura et al. 1999; Yoshida et al. 1996). E_2_, in turn, is the main oestrogen in birds, which regulates ovarian development and the expression of genes related to sexual differentiation (Rosati et al. 2021). In addition, it influences the expression of aromatase, creating a positive feedback that reinforces sexual differentiation in chickens (Nakabayashi et al. 1998). It has also been reported that meiotic germ cells have oestrogen receptors, reinforcing the role of this hormone in both gonadal differentiation and the maintenance of reproductive physiology (Carreau et al. 2001).

Therefore, understanding the formation and functioning of the reproductive system during embryonic development is fundamental, especially when it comes to commercially bred animals, as it facilitates the application of reproductive biotechniques. In the present study, we investigated the immunolabelling of P450c17, aromatase P450 and oestrogen receptor alpha in rhea embryos during critical stages of gonadal differentiation (Days 9, 12, 15, 18, 21 and 24 of incubation). Understanding the interactions between these molecules may contribute to advancing knowledge about the reproductive biology of *R. americana* , with potential applications in *ex situ* management and conservation programmes.

## 2 Materials and Methods

### 2.1 Ethical Aspects and Origin of Animals

This project was approved by the Ethics Committee for the Use of Animals (CEUA) under the number 6890081220 and by the Biodiversity Authorization and Information System (SISBIO) under the number 85407‐1. Greater rhea eggs were obtained from CEMAS—Centro de Multiplicação de Animais Silvestres (Wild Animal Multiplication Center) at the Universidade Federal Rural do Semi Árido (UFERSA).

### 2.2 Experimental Design and Collection of the Embryos/Foetuses

One‐day‐old eggs were used and incubated artificially at 38°C and 80%–90% humidity. From the second day of incubation, a period of 9–24 days was considered. During this incubation period, three embryos were collected every 3 days (D9, D12, D15, D18, D21, D24), totalling 18 individuals.

To euthanize the embryos, the pole of the egg air chamber was opened and absorbent cotton soaked in a lethal dose of isoflurane was inserted. The embryos or fragments containing the genital organs were fixed in 4% paraformaldehyde for subsequent histological processing. The D9 samples were processed in their entirety due to their small size (approximately 1 cm), making dissection impossible. At the other ages, the mesonephric‐renal region with the gonad was isolated for processing.

### 2.3 Histological Processing

The samples were processed in an adapted way, based on the methodology of Junqueira and Carneiro (2013). Dehydration was carried out in ethyl alcohols of increasing concentrations (70%–100%) for 24 h, followed by diaphonization with two xylene baths (1 h 30 min each) and inclusion in histological paraffin (60°C, two 2‐h changes). The blocks were sectioned at a thickness of 6 μm.

### 2.4 Immunohistochemistry

Sections of the embryo samples were mounted on positively charged slides and processed for immunolabelling of cytochrome P450c17, P450 aromatase and ERα. Antigen recovery was carried out by incubating the slides in a recovery buffer (low pH, K8005; Dako, Santa Clara, CA, USA) for 20 min at 98°C, using a pressure cooker. For the other blocking reactions, the Rabbit specific HRP/DAB Detection IHC Detection Kit—Micro‐polymer (ab236469; Abcam Inc., Cambridge, MA, USA) was used, following the manufacturer's recommendations.

However, to immunolabel the proteins, the slides were incubated for 30 min with primary antibodies anti‐Cytochrome P450 17A1/CYP17A1 (1:250—ab231794), anti‐Aromatase (1:200—ab18995), anti‐Oestrogen Receptor Alpha (1:150—ab3575) (Abcam Inc., Cambridge, MA, USA). Counterstaining was carried out with haematoxylin for 1 min and ammonia solution (0.5%) for 30 s. Fragments of testicles and uterine tubes from mice were used as positive and negative controls.

Immunostaining was observed using an optical light microscope (Olympus BX61VS). The assessment was made qualitatively using the H‐Score, defined as: [H‐score = ∑*P*_*i*_ (*i* + 1)], adapted from the methodology of Wiweko et al. (2019) and Gomes et al. (2022). The intensity of the staining was based on a scale ranging from 0 to 3 (0, absent; 1, weak; 2, moderate; 3, strong), and the H score ranges from 0 to 300 (0, no staining; 1–150, weak score; 151–201, moderate score; 202–301, strong score; 301, maximum score). *P*_*i*_: Percentage of immunopositive cells; *i*: Staining intensity. The area demarcated for evaluation was 11,300 μm^2^ (for days D9 and D12) and 200,000 μm^2^ (other ages).

### 2.5 Statistical Analysis

The data were gathered and compiled using Microsoft Excel software, and then described statistically as the average score value. Due to the sample size, the variables were presented qualitatively.

## 3 Results

### 3.1 P450c17 Immunostaining

**FIGURE 1:**
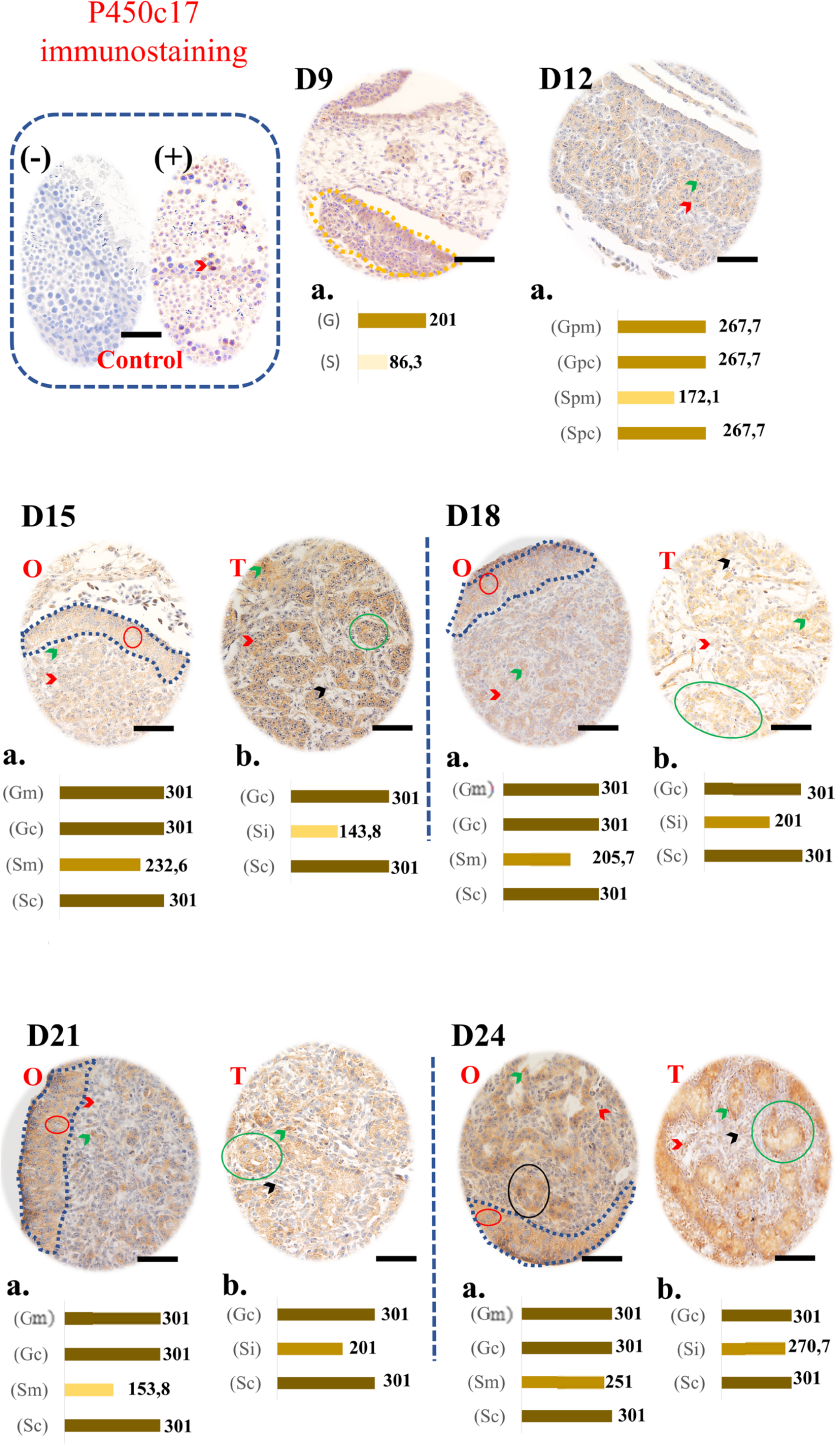
Immunohistochemistry for P450c17 in the developing gonads of Greater Rhea. The brown marking indicates the presence of the protein, with intensity varying from light to intense, seen illustratively in the graphs (‘a’ and ‘b’) for each day of development. Marked structures: Gonadal crest (D9, dashed yellow), cortex (dashed blue), germ cells (red circles), somatic cells (red arrowheads), primary sex cords (green circles), interstitial cells (black arrowhead) from D15 onwards, cytoplasmic granules (green arrows) and ovarian gaps (black circles). O, ovary; T, testis. Graphics: *D9* (G, germ cells; S, somatic cells); *D12* (Gpm/Gpc and Spm/Spc: Germ cells and somatic cells of the precursor structure of the medulla/cortex, respectively); *D15 to D24* [Gm/Gc and Sm/Sc (Ovary): Germ cells and somatic cells of the medulla/cortex; Gc/Sc and Si (Testis): Germ/somatic cells of the seminiferous tubule and somatic cells of the interstitium]. The negative control has no markings (control box). Magnification: 40×. Scale bars: 250 μm.

Representative images of the immunostaining and graphs of P450c17 enzyme staining intensities can be seen in Figure 1. P450c17 immunoreactivity was cytoplasmic in germ cells and characterised by the presence of granules in the cytoplasm of somatic cells (green arrow). On D9, the germ cells of the gonadal crest showed a moderate score (201), while the somatic cells showed a weak score (86.3).

At D12, both germ cells and somatic cells in the cortical region had a strong score (267.7), while in the medullary region the somatic cells had a moderate score (172.1).

From D15 to D24, germ cells and somatic cells in the cortical region (ovary) and seminiferous tubule (testis) had a strong score (301). The somatic cells of the medulla and interstitium maintained a moderate score, except for the interstitial cells of D24, which had a strong score (270.7). Marking was also observed in the lumen of the seminiferous tubules and in ovarian lacunae.

### 3.2 Aromatase Immunostaining

**FIGURE 2:**
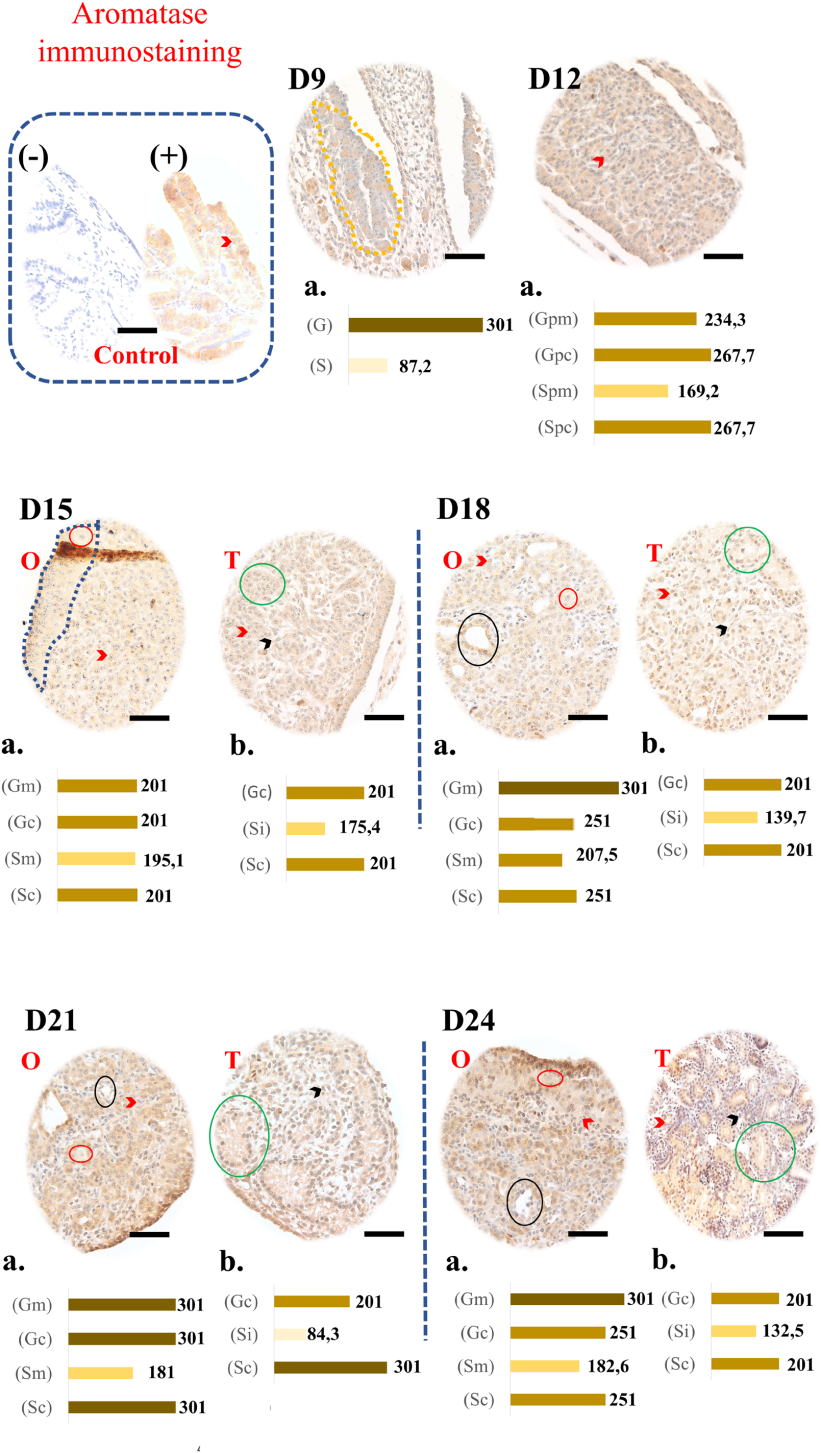
Immunohistochemistry for aromatase in the forming gonads of embryos of Greater Rhea. The brown marking indicates the presence of the protein, with intensity varying from light to intense, seen illustratively in the graphs (‘a’ and ‘b’) for each day of development. Marked structures: Gonadal crest (D9, dashed yellow), cortex (dashed blue), germ cells (red circles), somatic cells (red arrowheads), primary sex cords (green circles), interstitial cells (black arrowhead) from D15 onwards and ovarian gaps (black circles). O, ovary; T, testis. Graphics: *D9* (G, germ cells; S, somatic cells); *D12* (Gpm/Gpc and Spm/Spc: Germ cells and somatic cells of the precursor structure of the medulla/cortex, respectively); *D15 to D24* [Gm/Gc and Sm/Sc (Ovary): Germ cells and somatic cells of the medulla/cortex; Gc/Sc and Si (Testis): Germ/somatic cells of the seminiferous tubule and somatic cells of the interstitium]. Unmarked negative controls (control box). Magnification: 40×. Scale bars: 250 μm.

Representative images of the immunostaining and graphs of the aromatase enzyme staining intensities are shown in Figure 2. Aromatase showed diffuse or granular cytoplasmic markings. At D9, the germ cells showed a strong score (301), while the somatic cells had a weak score (87.2).

At D12, the cortical region showed a strong score (267.7) for both cell types, while in the medullary region, the germ cells showed a moderate score and the somatic cells showed a weak score.

In the D15 ovary, all cell types had a moderate score. From D18 onwards, germ cells in the medulla (especially in lacunae) reached a maximum score (301), while somatic cells varied between moderate scores (181–207.5). Cortex cells had a strong score: 251 (D18), 301 (D21) and 251 (D24).

In the testes, extracellular labelling was observed in the lumen of the primary sex cords. The cells of the seminiferous tubules had a predominant score of 201 (moderate), except in D21 (301). The interstitial cells had a moderate score on D15 (175, 4). And weak on the other days: 139.7 (D18), 84.3 (D21), 132.5 (D24).

### 3.3 ERα Immunostaining

**FIGURE 3:**
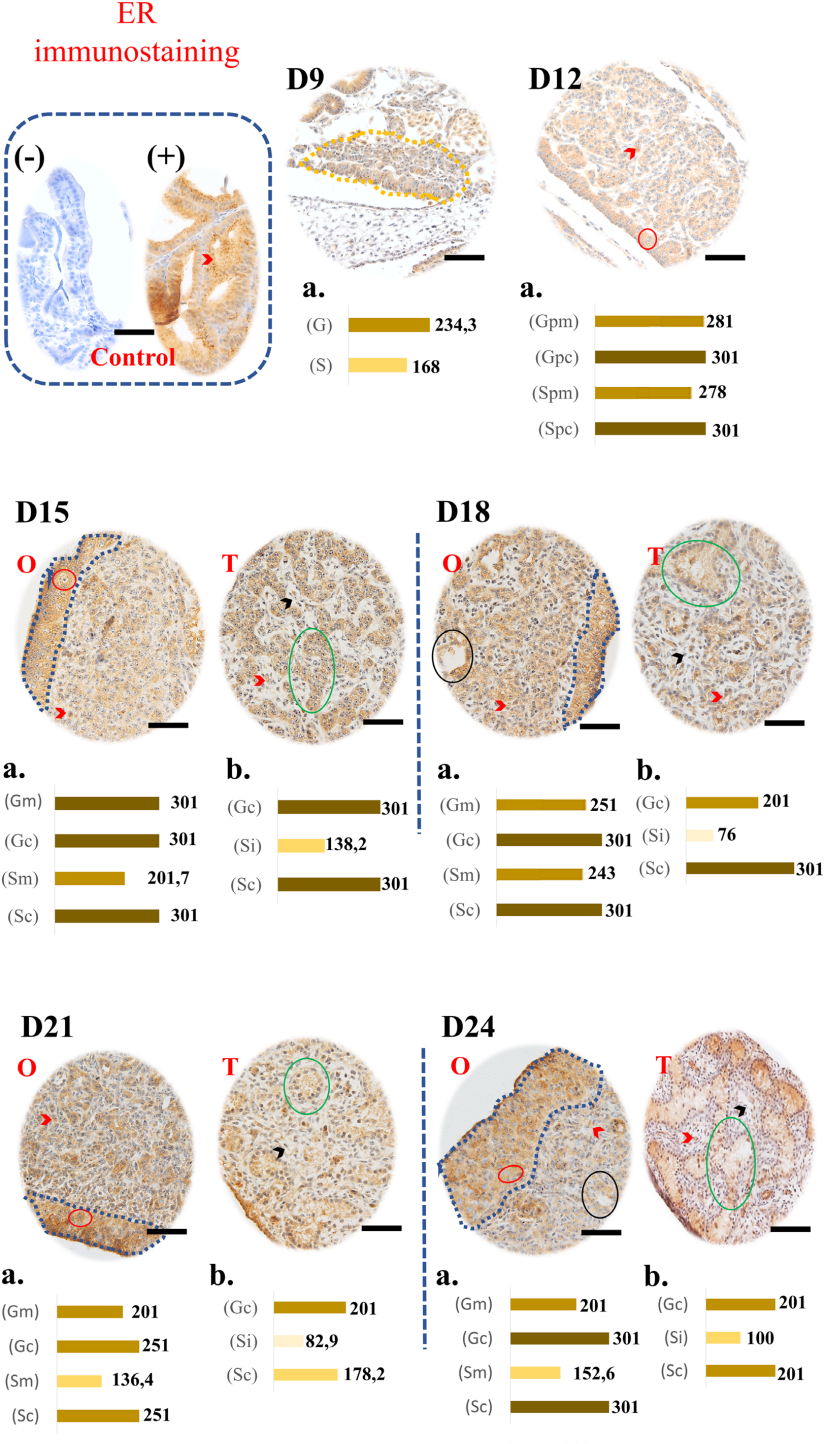
Immunohistochemistry for ERα in gonads of developing Greater Rhea embryos. The brown marking indicates the presence of the protein, with intensity varying from light to intense, seen illustratively in the graphs (‘a’ and ‘b’) for each day of development. Marked structures: Gonadal crest (D9, dashed yellow), cortex (dashed blue), germ cells (red circles), somatic cells (red arrowheads), primary sex cords (green circles), interstitial cells (black arrowhead) from D15 onwards and ovarian gaps (black circles). O, ovary; T, testis. Graphics: *D9* (G, germ cells; S, somatic cells); *D12* (Gpm/Gpc and Spm/Spc: Germ cells and somatic cells of the precursor structure of the medulla/cortex, respectively); *D15 to D24* [Gm/Gc and Sm/Sc (Ovary): Germ cells and somatic cells of the medulla/cortex; Gc/Sc and Si (Testis): Germ/somatic cells of the seminiferous tubule and somatic cells of the interstitium]. Negative control without labelling (control box). Magnification 40×. Scale bars: 250 μm.

Representative images of the immunostaining and graphs of the ERα staining intensities are presented in Figure 3. For ERα, cytoplasmic, nuclear and extracellular markings were observed. At D9, germ cells showed a strong score (234.3) and somatic cells a moderate score (168). At D12, all cell types showed a strong score, with maximum values in the cortical region.

In the ovaries, cortical cells maintained a maximum score until D24, except at D21 (251). Germ cells in the medulla showed a downward trend in the score over time: (D15:301; D18:251; D22:201; D24:201). In medullary somatic cells, the moderate score ranged from 201.7 (D15) to 152.6 (D24). Ovarian lacunae showed intense markings.

In the testes, cortex cells showed a change in score (301 on D15 to 201 on D24), with a tendency to decrease. Interstitial cells maintained a weak score throughout the period. Marking was also observed in the forming lumen.

## 4 Discussion

The majority of studies on sexual differentiation and gonadal development in birds use the chicken as an experimental model (Smith and Sinclair 2004), and investigations into ratites are rare. As a pioneer, our group recently characterised the embryonic development of rheas and determined the time window for sexual differentiation (Cristofoli et al. 2025). Based on these findings, the present study sought to deepen our understanding of the immunolabelling of the steroidogenic enzymes P450c17, aromatase P450, and the oestrogen receptor alpha (ERα) in germ cells and somatic cells throughout the gonadal embryogenesis of rheas.

We identified P450c17 labelling in ovarian germ cells, suggesting possible involvement in local androgen biosynthesis. This protein showed a strong score in germ cells and their adjacent somatic cells from D12 to D24, precisely from the beginning of the time window of sexual differentiation of rheas of both sexes, presented in our preliminary study (Cristofoli et al. 2025). P450c17 is an important enzyme in the steroidogenic pathway that acts in the synthesis of androgens essential for the biosynthesis of sex steroid precursors, through the conversion of pregnenolone and progesterone into their corresponding androgenic products, dehydroepiandrosterone (DEHA) and androstenedione (Conley et al. 1995; Payne and Hales 2004; Burris‐Hiday and Scott 2021). Thus, it is believed to be involved in the formation of four of the main steroid hormones, namely testosterone, estradiol, cortisol and DEHA (Hall 1986; Miller 2002). In bird embryos, male and female gonadal differentiation and development depend on the presence or absence of enzymes, such as P450c17 and aromatase (Nishikimi et al. 2000; Jin et al. 2020). Yoshida et al. (1996) first detected P450c17 mRNA on Days 5–6 of incubation in some male and female embryos in *Gallus gallus* . Additionally, Nishikimi et al. (2000) showed that, in females, gonadal expression of P450c17 mRNA increases gradually during the period of 5–8 days of incubation, while in males it increases sharply on the 6th day of incubation. The authors suggest that these increases may be reflected in the levels of enzymatic activity in the gonad. Considering that the duration of embryonic development in *Gallus* sp. and *Rhea* sp. is 21 and 42 days, respectively, and that the periods mentioned for both chickens, 5–8 days of incubation, and rheas, 12–15 days, are considered the time window for gonadal differentiation, we can deduce that the levels of P450c17 enzyme activity in rheas are also related to this event.

Our findings also show that both germ cells and somatic cells were marked for P450c17. In addition, the score of ovarian medullary somatic cells remained moderate throughout the study period. However, in testicular interstitial cells, the D24 score was robust. Liu, Yao, Bendavid, et al. (2005) concluded in their study that P450c17 is present in the germ cell lineage of the testis, raising the possibility that both germ cells and mature sperm have the ability to form androgens from pregnenolone and progesterone, or that this enzyme may have alternative functions that affect sperm formation. Furthermore, it has been suggested that P450c17 may play additional catalytic roles, such as squalene monooxygenase activity, critical in cholesterol biosynthesis, involving crucial steps in sperm formation, function and fertility (Lieberman and Warne 2001; Liu, Yao, and Papadopoulos 2005). Thus, we believe that these facts may be similar for the ovary, justifying its presence in germ cells of this organ. In the interstitial compartment of the testis, there are various cell types, including Leydig cells, which are steroidogenic and essential for the morphogenesis of the foetal testis. With this, the change in score, indicated by a strong intensity of P450c17 at D24 in the interstitium, may be an expected event, since it is the main production site to promote spermatogenesis, as well as the regulation of the spermatogonial stem cell niche and foetal testicular differentiation (Heinrich and DeFalco 2020).

Aromatase has been strong in germ cells since D9, especially in the ovarian cortical and medullary regions between D18 and D24. Aromatase is a microsomal enzyme, located in the smooth endoplasmic reticulum, which is part of an enzyme complex that includes cytochrome P450 aromatase, a product of the CYP19A1 gene, and an NADPH‐dependent cytochrome P450 reductase, known as a ubiquitous flavoprotein (Guiguen et al. 2010; Rosati et al. 2021; Simpson et al. 1994). Cytochrome P450 aromatase is essential for the irreversible conversion of testosterone into estradiol‐17β (Rosati et al. 2021). According to some studies in chickens, the expression of *P450arom mRNA* can occur in the female gonad around Day 6, being associated with sexual differentiation, increasing the production of oestrogens, which can promote cell proliferation of the left gonad (Nishikimi et al. 2000; Nomura et al. 1999; Yoshida et al. 1996; Mfoundou et al. 2021). In addition, Nakabayashi et al. (1998) also showed that aromatase transcripts, in *Gallus* sp., were detected in the left and right gonads of the female embryo on the 7th day of incubation and that the expression of this gene continued until the 14th day of incubation. Thus, similar to chickens, the extensive presence of aromatase in female rheas, observed in this study, may also be playing an essential role in the determination and formation of the ovary, promoting the synthesis of oestrogen and the expression of its receptor in the left gonad, which consequently results in the development of the functional left ovary (Bruggeman et al. 2002; Lambeth et al. 2016).

Our study also reported the presence of gaps in the ovarian medulla, which appeared from D18 onwards, with a maximum score. Jin et al. (2020) also reported robustly expressed aromatase positivity in chicken ovarian medulla. According to the study by Mizia et al. (2023) in chickens, from 9 days of incubation, it is also possible to observe lacunae, that is cavities in the ovarian medulla, which become larger in subsequent stages. These authors also describe that the left gonad develops into a typical ovary with a medulla containing medullary cords with singular germ cells, which become lacunae. Komárek and Procházková (1970) consider the lacunar zone to be an expansion chamber for the growing follicles, as well as playing a role as diffusion chambers in the supply of nutritional substances and the elimination of waste from the follicles. In addition, lacuna cells can also help to absorb and eliminate large quantities of vitelline from ruptured follicles in the chicken ovary (Nili and Kelly 1996; Mizia et al. 2023). Thus, we believe that the robust presence of aromatase is linked not only to sexual differentiation in rheas, as discussed above, but also to the complete development of the ovary, including a contribution to the formation of ovarian follicles, which until D24 has not yet been observed in rheas.

In the testis, moderate aromatase labelling in the seminiferous cords and weak labelling in the interstitial cells of the D18–D24 shows its involvement in the regulation of spermatogenesis. Although some older studies present data showing the absence of aromatase during male embryonic development (Nishikimi et al. 2000; Smith et al. 1997), it is now known that this enzyme plays a central role in the aromatase‐oestrogen system, functioning as a critical regulator of spermatogenesis and spermiogenesis in vertebrates (Rosati et al. 2021, 2020). Aromatase and P450c17 transcripts are already present in the gonads of avian embryos even before gonadal differentiation, showing that the steroidogenic system is active early on (Bruggeman et al. 2002; Nomura et al. 1999; Yoshida et al. 1996; Liu et al. 2025). There are reports that testicular germ cells and spermatozoa in the epididymis act as sites of oestrogen synthesis, suggesting their role as regulators or modulators of the testicular and epididymal germ epithelium, reinforcing the relevance of oestrogens in avian reproductive physiology (Kwon et al. 1995). In rat testis, aromatase can also be immunolocalized in germ cells, progressively decreasing as they mature (Carreau et al. 2001). However, in addition to testicular germ cells, this enzyme is present in Leydig cells, which also synthesise oestrogen (Rosati et al. 2021, 2020). Thus, we show that aromatase plays a physiological role in the maintenance of male gonadal functions in emus, directly involving germ cells and/or through Leydig cells.

In addition, the present study also focused on oestrogen receptor alpha (ERα), which is involved in the differentiation of the gonads and müllerian ducts (Mattsson and Brunström 2017). ERα was robustly present in germ cells from D9, with intense labelling in the ovary and a tendency to decrease in the medulla from D21 onwards. During embryogenesis in birds, the gonads of both sexes acquire the ability to respond to oestrogens, even before morphological differentiation (Smith et al. 1997; Jin et al. 2020). Oestrogen receptor *mRNA* is expressed in the gonads of both sexes before differentiation (E4) and persists after the onset of sexual dimorphism (E7) (Andrews et al. 1997). These studies corroborate our findings on the presence of ERα at D9 in emus, as it is known that oestrogens act through the high‐affinity nuclear receptors ERα and ERβ, exerting complementary or opposite physiological functions, regulating gene expression (Mattsson and Brunström 2017); however, ERα is the main mediator of oestrogenic action (Zhao et al. 2017). As observed in our findings, other studies have also reported that ER is present in the germinal epithelium of the left ovary and medulla in birds (Andrews et al. 1997; Nakabayashi et al. 1998; Yoshida et al. 1996). In addition, it has been reported that during the embryonic development of Japanese quails, the ovaries produce high amounts of oestrogen (Balthazart et al. 2009). Thus, these studies, added to our findings, further reinforce the essential role of oestrogens in the embryonic development of emus, actively participating in female gonadogenesis through the action of ERα.

According to Smith et al. (1997), oestrogen synthesis is a specific characteristic of females and only occurs at the time of gonadal differentiation. However, as previously discussed, here we reinforce the presence of aromatase (responsible for oestrogen synthesis) in emu testes and the relevance of oestrogen for regulating and modulating the testicular germinal epithelium in chickens (Rosati et al. 2021, 2020). Thus, it makes sense to observe the presence of ERα during emu embryonic development, as reported in this study, with a moderate (mainly at D22 and D24) and weak (D15–D24) score in the seminiferous cord and interstitium regions, respectively. Nakabayashi et al. (1998) showed that the oestrogen receptor gene is expressed in the cortex of females and has temporary expression limited to the early stages of development in males. Although these findings are still the subject of debate, growing evidence indicates that oestrogens act throughout the male genital tract via these receptors in rats (Carreau et al. 2006, 2001; Hess et al. 2021). The same research group has shown that in spermatogenesis, the regulation of the number of germ stem cells and the maturation of spermatids are influenced by these hormones (Carreau et al. 2001). In addition, Rosati et al. (2021) also reported that, in birds, oestrogens act synergistically with testosterone in promoting spermatogenesis. In addition to these interactions, Bruggeman et al. (2002) had associated AMH with a negative feedback with estradiol and vice versa, promoting the inhibition of cortical development of the testis, leading to medullary formation and the regression of both müllerian ducts in males. This suggests an important role for oestrogens in male reproductive physiology in emus, reinforcing the autocrine and paracrine role of testicular cells in testicular morphogenesis. However, the regulation of ER expression by oestrogens in these testes still needs to be investigated.

## 5 Conclusion

This study demonstrated the expression of P450c17, aromatase and ERα in the gonads of developing rhea's embryos (up to the 24th day of incubation), especially during the window of gonadal dimorphism. The differences in marking intensity between germ cells and somatic cells suggest that these factors act differently in the cellular compartments. P450c17, aromatase and ERα were also expressed at relevant levels, with aromatase standing out in the ovarian lacunae. These findings suggest that the interaction between these factors regulates not only sexual differentiation, but also the formation of the functional left ovary and the organisation of the ovarian medulla, fundamental stages for folliculogenesis, not yet evident until D24. In the testis, the presence of these molecules reinforces their importance in morphogenesis and regulation of the spermatogonial niche. These results provide support for future research into steroidogenesis, hormonal regulation and possible sex reversal mechanisms in ratitas, as well as contributing to the improvement of reproductive management and conservation strategies for *R. americana* .

## Author Contributions

Conceptualization: Antônio Chaves de Assis Neto, Moacir Franco Oliveira and Marilú Cristofoli; Formal analysis: Marco Aurélio Schiavo Novaes; Funding acquisition: Antônio Chaves de Assis Neto; Investigation, methodology and validation: Marilú Cristofoli, Danielle Cristina Calado de Brito, Mayla Magalhães de Oliveira Alcobaça and Moacir Franco Oliveira; Supervision: Antônio Chaves de Assis Neto; Writing – original draft: Marilú Cristofoli and Danielle Cristina Calado de Brito; Writing – review and editing: Marilú Cristofoli, Danielle Cristina Calado de Brito, Mayla Magalhães de Oliveira Alcobaça, Flavia Maria Pia Montenegro Donoso, Fabiana Morse Gosson Jorge, Moacir Franco Oliveira and Antônio Chaves de Assis Neto.

## Funding

This study was financed in part by the Coordenação de Aperfeiçoamento de Pessoal de Nível Superior—Brasil (CAPES)—Finance Code 001.

## Conflicts of Interest

The authors declare no conflicts of interest.

## Data Availability

The data that support the findings of this study are available upon request to the corresponding author.
